# Pathologically decreased expression of miR-193a contributes to metastasis by targeting WT1-E-cadherin axis in non-small cell lung cancers

**DOI:** 10.1186/s13046-016-0450-8

**Published:** 2016-11-07

**Authors:** Junjie Chen, Shenmeng Gao, Chunjing Wang, Zhonggai Wang, Huxiang Zhang, Kate Huang, Bin Zhou, Haiying Li, Zhijie Yu, Jianbo Wu, Chengshui Chen

**Affiliations:** 1 Department of Respiration, The First Affiliated Hospital of Wenzhou Medical University, Nanbaixiang, Ouhai District, Wenzhou, 325000 Zhejiang Province China; 2 Laboratory of Internal Medicine, The First Affiliated Hospital of Wenzhou Medical University, Nanbaixiang, Ouhai District, Wenzhou, Zhejiang Province China; 3 School of Laboratory Medicine & School of Life Science, Wenzhou Medical University, Nanbaixiang, Ouhai District, Wenzhou, Zhejiang Province China; 4 Pathology Department, The First Affiliated Hospital of Wenzhou Medical University, Nanbaixiang, Ouhai District, Wenzhou, Zhejiang Province China

**Keywords:** MiR-193a, Wilm’s tumor-1, E-cadherin, Epithelial-to-mesenchymal transition

## Abstract

**Background:**

The metastatic cascade is a complex and multistep process with many potential barriers. Recently, miR-193a has been reported to be a suppressive miRNA in multiple types of cancers, but its underlying anti-oncogenic activity in non-small cell lung cancers (NSCLC) is not fully elucidated.

**Methods:**

The expressions of miR-193a (miR-193a-5p) in human lung cancer tissues and cell lines were detected by real-time PCR. Dual-luciferase reporter assay was used to identify the direct target of miR-193a. Cell proliferation, apoptosis, and metastasis were assessed by CCK-8, flow cytometry, and Transwell assay, respectively.

**Results:**

The expression of miR-193a in lung cancer tissues was decreased comparing to adjacent non-tumor tissues due to DNA hypermethylation in lung cancer tissues. Ectopic expression of miR-193a inhibited cell proliferation, colony formation, migration, and invasion in A549 and H1299 cells. Moreover, overexpression of miR-193a partially reversed tumor growth factor-β1 (TGF-β1)-induced epithelial-to-mesenchymal transition (EMT) in NSCLC cells. Mechanistically, miR-193a reduced the expression of WT1, which negatively regulated the protein level of E-cadherin, suggesting that miR-193a might prevent EMT via modulating WT1-E-cadherin axis. Importantly, knockdown of *WT1* resembled the anti-cancer activity by miR-193a and overexpression of *WT1* partially reversed miR-193a-induced anti-cancer activity, indicating that WT1 plays an important role in miR-193a-induced anti-cancer activity. Finally, overexpression of miR-193a decreased the growth of tumor xenografts in mice.

**Conclusion:**

Collectively, our results have revealed an important role of miR-193a-WT1-E-cadherin axis in metastasis, demonstrated an important molecular cue for EMT, and suggested a therapeutic strategy of restoring miR-193a expression in NSCLC.

**Electronic supplementary material:**

The online version of this article (doi:10.1186/s13046-016-0450-8) contains supplementary material, which is available to authorized users.

## Background

Lung cancer is the leading cause of cancer-related deaths in the world [1]. Although great efforts have been made toward improving overall survival and reducing cancer-related death including discovering of new biomarkers [2], development of specific drugs [3], and establishment of modified operation [4], the outcome is still poor. Metastasis is the leading reason for the resultant mortality in more than 90 % of patients with cancer, including lung cancer. Metastasis is a complex process in which the metastatic potential of NSCLC cells is influenced by cell-intrinsic identities and extrinsic microenvironment factors. Metastasis is also associated with epithelial-mesenchymal transition (EMT), a biologic process during which epithelial cells acquire new features of mesenchyme [5]. The reduction of E-cadherin, a marker of epithelial cells, is currently thought to promote metastasis during early carcinogenesis progression [6]. E-cadherin is inhibited by many transcript factors such as *Snail*, *ZEB1/2*, and *Slug*, which are induced by multiple signaling pathways including Wnt, TGF-β, and Notch [7, 8]. For example, TGF-β promotes tumor progression through enhancing migration and invasion, and induces EMT through inhibiting the expressions of *Snail* and *Slug* [9].

Wilms tumor 1 gene (WT1) was firstly identified as a tumor suppressor gene, encoding a 49–52 kDa protein with four zinc fingers in C-terminal domain in nephroblastoma, also known as Wilms tumor, a childhood tumor of the kidney [10]. However, subsequent accumulating studies demonstrated that high expression of *WT1* was detected in different types of solid cancers and hematological malignancies, such as breast cancer [11], lung cancer [12], and leukemia [13]. At least four major isoforms of *WT1*, including 17AA(+)KTS(+), 17AA(+)KTS(-), 17AA(-)KTS(+), and 17AA(-)KTS(-), were discovered according to two different sites. These isoforms were expressed in primary solid tumors and primary leukemic blasts and presented different functions [14]. Furthermore, overexpression of WT1 enhanced proliferation through upregulation of cyclin D1 and p-pRb in NSCLC cells [12]. WT1 suppressed the expression of E-cadherin through directly binding its promoter to enhance the invasion [15]. Thus, WT1 plays an important role in promoting proliferation and invasion in NSCLC cells.

MicroRNAs (miRNAs) are a class of short single-stranded noncoding RNAs with approximately 20 nucleotides in length. MiRNAs inhibit the target gene expressions by either inhibiting translation or inducing mRNA degradation [16]. Recently, emerging data have shown that miRNAs were often deregulated in multiple types of cancers and played an important role in proliferation, apoptosis, differentiation, drug resistance, and metastasis [17–20]. In NSCLC, only a few miRNAs were identified in metastasis, including miR-31 [21] and miR-135b [22]. Thus, it is an urgent need to identify useful miRNAs that provide as a potential new therapeutic arget. However, whether WT1 can be regulated by miRNAs in lung cancer cells is not fully understood.

MiR-193a was reported to be a suppressive miRNA and overexpression of miR-193a suppressed proliferation and promoted apoptosis via targeting several oncogenes, such as *c-KIT* [23] and *MCL-1* [24]. In addition, miR-193a regulated metastasis in solid cancers including NSCLC. For example, miR-193a inhibited invasion by negatively regulating ERBB4/PIK3R3/mTOR/S6K2 signaling pathway in NSCLC [25]. MiR-193a inhibited the metastasis of lung cancer cells by deregulating the expression of tumor-related proteins [26]. Thus, these results suggest that miR-193a might regulate the metastasis in NSCLC. The decrease of E-cadherin is an important procedure for the promotion of invasion. However, whether miR-193a can regulate E-cadherin expression is not determined. Because WT1 is implicated in the metastasis of NSCLC through inhibiting the expression of E-cadherin [15], we hypothesized that one mechanism of anti-metastasis activity of miR-193a might perform by modulating WT1-E-cadherin axis.

Here, we report a miR-193a-WT1-E-cadherin axis in NSCLC. Decreased expression of miR-193a regulated TGF-β1-induced EMT progress. Overexpression of miR-193a inhibited migration and invasion via modulating WT1-E-cadherin axis. Additionally, miR-193a partially prevented TGF-β1-induced EMT, suggesting that miR-193a plays an important role in TGF-β1-induced EMT. Therefore, targeting miR-193a-WT1-E-cadherin axis might provide a novel strategy to improve survival in lung cancer patients.

## Methods

### Cell lines and tissue specimens

Multiple lung cancer cell lines and normal lung epithelial cell line BEAS-2B (Cell Bank of Shanghai Institutes for Biological Sciences, Shanghai, China) were used in this study. A549 and H1299 were cultured in RPMI 1640 medium, whereas 293T was cultured in Dulbecco’s Modified Eagle Medium (DMEM) high-glucose medium. All cells were supplemented with 10 % fetal bovine serum (Invitrogen, Carlsbad, USA) and maintained in a humidified 37 °C incubator with 5 % CO_2_. Total 62 paired lung cancer specimens including lung cancer and paired adjacent normal tissues were collected from patients undergoing surgical resection in the Department of Thoracic Surgery, the First Affiliated Hospital of Wenzhou Medical University. Non-tumor samples from the macroscopic tumor margin were isolated at the same time and used as the matched adjacent normal tissues. Informed consents were obtained from all patients. All the samples were divided into two parts. One part was immediately frozen and stored in liquid nitrogen until RNA extraction. Another part was stored in formalin for pathology analysis. These patients’ histological type was further performed by an experienced pathologist using standard hematoxylin and eosin staining and the staging of NSCLC by a clinical oncologist according to the International Association for the Study of Lung Cancer (IASLC) TNM-classification. Clinicopathological characteristics of the NSCLC patients were shown in Additional file 1: Table S1. Adjacent tissue was located within 3 cm of the edge of the tumor tissue. This study was approved by the Research Ethnics Committee of Wenzhou Medical University. 5′-azacytidine (AZA, Sigma-Aldrich, St Louis, MO, USA) was dissolved in distilled water and kept at -20 °C until used.

### Cell proliferation assay (Cell Counting Kit-8)

Lung cancer cells transfected with pLVX-miR-193a or pLVX-NC were seeded into 96-well plates (6.0 × 10^3^ cells per well). Cell proliferation was assessed by Cell Counting Kit-8 assay (Beyotime Institute of Biotechnology, Shanghai, China). Cells were added with 10 μl CCK-8 and incubated for 2 h at 37 °C. The absorbance of each well was read on a spectrophotometer at 450 nm. Three independent experiments were performed in quintuplicate.

### Plasmid construction

To construct the plasmid that expresses miR-193a in mammalian cells, the primary sequence of has-pre-miR-193a and its flanking regions were amplified by specific primer pairs and then were cloned into lentivirus vector pLVX-puro (Clontech, Palo Alto, USA). The whole coding sequence (CDS) of WT1 isoforms (NM_000378) were directly synthesized (Genewiz, Suzhou, China) and then cloned into retrovirus pMSCV-puro vector (Clontech). Human *WT1* CDS including the predicted miR-193a target sites was amplified by PCR and cloned into pMIR-REPORT vector named as pMIR-WT1CDS (Ambion, Dallas, USA). The mutation on miR-193a-binding sites in human *WT1* CDS was generated by the site-directed mutagenesis kit (Agilent Technologies, Palo Alto, USA). All the primer sequences were shown in Additional file 2: Table S2. All of these constructs were confirmed by sequencing.

### Retrovirus production and cell transfection

HEK293T cells (4 × 10^6^) were plated in 10 cm dish. After 24 h, LVX-puro-miR-193a and MSCV-puro-WT1 vectors were cotransfected with packaging and envelope vectors into HEK293T cells. Virus was harvested from the supernatant at 48 h posttransfection, and was mixed with 8 μg/ml polybrene (Sigma-Aldrich, St. Louis, USA) to increase the infection efficiency. To select the cells stably expressing miR-193a, 2 μg/ml puromycin (Medchemexpress, Princeton, USA) was added into the supernatant for 1 week.

### Luciferase activity

A549 cells were seeded in 24-well plates at a density of 1.0 × 10^5^ cells per well. After growth for 24 h, each well was transiently cotransfected with 100 ng pMIR-REPORT plasmid containing 10 ng internal control vector pRL-SV40 (Promega, Madison, USA), wild-type or mutant pMIR-WT1CDS, and 60 pmol scramble or miR-193a mimics using Hiperfect transfection reagent (Qiagen). Firefly and renilla luciferase activities were measured in cell lysates after transfection for 24 h by the Dual-Luciferase Reporter Assay System (Promega). The value of relative luciferase activity indicates the firefly luciferase activity normalized to that of renilla for each assay.

### RNA interference

Gene-specific short hairpin RNA (shRNA) for WT1 were designed and cloned into pSIREN-RetroQ (Clontech) retroviral vector. Control shRNA is a nonfunctional construct provided from Clontech. The sequences of sh-WT1 were indicated in Additional file 2: Table S2. These shRNAs vectors or negative vector were co-transfected with packaging plasmids Gap-pol and VSV-G into 293T cells to produce retrovirus. Supernatants containing retrovirus were collected at 48 h after transfection and were used to infect lung cancer cells. Positive clones with stable knockdown of WT1 were selected by 2 μg/ml puromycin (Medchemexpress) for 1 week.

### RNA extraction and stem-loop reverse transcription-polymerase chain reaction

Total RNA was extracted using Trizol reagent (Invitrogen, Carlsbad, CA) following the procedure by some slight modifications. The cells or tissues were incubated for five min at room temperature after being lysed with 1 ml Trizol reagent. The lysates were vigorously mixed with 300 μl chloroform and laid for 15 min at room temperature. After centrifugation for 20 min at 4 °C, 500 μl isopropanol was applied to precipitate RNA in the upper solution. Finally, RNA was dried and dissolved with RNase-free water. Mature miR-193a and U6 snRNA were reversely transcribed using stem-loop RT primer with miscript II RT Kit (Qiagen, Valencia, USA). Real-time PCR was performed using SYBR Green PCR Master Mix (Qiagen) in an Applied Biosystems 7500 instrument. Expression data were uniformly normalized to the internal control U6 and the relative expression levels were evaluated using the 2^-ΔΔCt^ method.

### Wound healing assay

Cells were pretreated with mitomycin (25 μg/ml, Medchemexpress), seeded in six-well plates, and cultured to confluence. Wounds of two-mm width were created with a plastic scriber, followed with washing away the floating cells with PBS. After incubation in a serum free medium for 12 h, cultures were observed and photos were taken under a microscope at 24 h.

### Immunofluorescence staining

Lung cancer cells were plated on a glass slide and fixed with 4 % formaldehyde for 30 min, followed with methyl alcohol for 20 min. Slides were blocked with 5 % bovine serum albumin for 2 h, and then were incubated for overnight with primary antibody for WT1, E-cadherin, Fibronectin, and Vimentin (Abcam, Cambridge, USA). Slides were washed and then incubated with second immunoglobulin-FITC or -PE antibody for 1 h and counterstained for nuclear by DAPI (0.2 μg/mL) for 15 min. The staining was analyzed under a fluorescence microscope (Olympus BX51, Tokyo, Japan) and Imstar FISH Progress software to capture the pictures.

#### Immunohistochemistry analysis

Frozen section-based immunohistochemistry analysis was performed to detect WT1 and E-cadherin expressions in xenografts from nude mice and human lung cancer tissues. Briefly, optimal cutting temperature-entrapped specimens were sliced as 4 μm sections and dried for 1 h at room temperature. The sections were treated with 3 % H_2_O_2_ in methanol for 10 min at room temperature to quench the endogenous peroxidase activity, followed by incubation with 5 % bovine serum albumin for 2 h at 37 °C to antagonize non-specific binding. The slides were incubated with primary antibodies, which were diluted with 1:100, for overnight, followed with further incubation with horseradish peroxidase-conjugated second antibody (Maixin, Fujian, China) at room temperature for 30 min. The tissue sections were immersed in 3.3′-diaminobenzidine and the reaction was terminated when positive staining was present. Subsequently, hematoxylin was used to counterstain the sections for 10 min at room temperature. Following dehydration and mount, the sections were visualized. The brown or sepia staining signal denotes positive regions. Image-Pro Plus software (v5.0) was used to analyze average values of integrated optical density (IOD) from five random fields per slide.

### Western blot analysis

Proteins extracted from cultured cells or tissues were quantitated by a protein assay (Beyotime, Shanghai, China). Cell or tissue lysates were fractionated by sodium dodecyl sulfate polyacrylamide gel electrophoresis and then transferred to nitrocellulose membrane (Millipore, Billerica, USA). Following blocking in PBS containing 5 % fat-free milk, the nitrocellulose membrane was incubated with primary antibodies against WT1, E-cadherin, Fibronectin, and Vimentin (Abcam). Blots were stripped and reprobed with β-actin antibody (Santa Cruz Biotechnology, California, USA) as an internal control. After overnight at 4 °C and then incubated with horseradish peroxidase-conjugated anti-rabbit IgG for 1 h at room temperature, the antigen–antibody immunoreactivity was detected in a sensitive digital imaging equipment (Bio-Rad, Richmond, USA) using a commercial ECL detection kit (Millipore). All experiments were repeated three times with the similar results.

### Migration and invasion assay

The migration and invasive ability of the cells was investigated using Transwells (Corning Costar Corp, Bedford, USA), which were put into 24-well plates. For migration assay, 5 × 10^4^ lung cancer cells were put into the upper compartment. For invasion assay, 1 × 10^5^ lung cancer cells were put into the upper compartment per well with the Matrigel-coated membrane (BD Biosciences, San Jose, USA), which was diluted with serum-free culture medium. In both assays, after 400 μl of RPMI 1640 containing 10 % fetal bovine serum was added to the lower compartment, lung cancer cells were suspended in 200 μl RPMI 1640 containing 2 % fetal bovine serum and were put into the upper compartment. After incubation for 48 h at 37 °C, the membrane inserts were removed from the plate, and non-invading cells were removed from the upper surface of the membrane. Cells that moved to the bottom surface of the chamber were fixed with 2 % paraformaldehyde for 20 min, and stained with 0.1 % crystal violet for 30 min. Then, the cells were imaged and counted in at least 10 random fields using a CKX41 inverted microscope (Olympus, Tokyo, Japan). The assays were conducted three independent times.

### Methylation-specific polymerase chain reaction (MSP)

Genomic DNA was extracted from lung cancer cell lines, lung cancer tissues, and adjacent non-tumor tissues using DNA Purification Kit (Qiagen). Treatment of DNA with bisulfite was performed by a commercially available kit (Millipore). The methylation-specific polymerase chain reaction (MSP) for promoter of pre-miR-193a was designed by MethPrimer software [27].

### Tumorigenicity in nude mice

Male athymic nude mice were purchased from SLAC (Shanghai SLAC Laboratory Animal, Shanghai, China). Animal-related experiments were performed according to the Guide for the Care and Use of Laboratory Animals (National Institutes of Health Publications) and approved by the committee for humane treatment of animals at Wenzhou Medical University Institutional Guidelines. The animals were maintained under specific pathogen free (SPF) conditions in the Animal Facility, Wenzhou Medical University. To determine whether miR-193a impacts tumorigenesis, 14 four-week-old mice were randomly divided into two equal groups. A total of 1 × 10^7^ viable A549-LVX-NC or A549-LVX-miR-193a cells were trypsinized and resuspended with 200 μl sterile 1× PBS and injected subcutaneously into right flank of each nude mouse. Both the long and short diameters of xenografts were measured using vernier calipers every 3 days after 2 weeks. All mice were sacrificed and the tumors from each mouse were harvested and weighed when the experiment was terminated at six weeks after tumor cell inoculation. Tumor volumes were measured using the equation V(mm^3^) = A × B^2^/2, where A is the largest diameter and B is the perpendicular diameter. Tumor lysates were prepared for western blot and tumors were isolated for standard immunohistochemistry analysis.

#### Statistical analysis

Data are presented as mean ± standard error. Generally, a two-tailed Student’s *t*-test was employed to evaluate the differences between groups. Differences are considered as significant when the *P* value is <0.05. All statistical analyses were performed with SPSS 22.0 software (SPSS Inc., lllinois, USA).

## Results

### Downregulation of miR-193a in lung cancer tissues

**Fig. 1:**
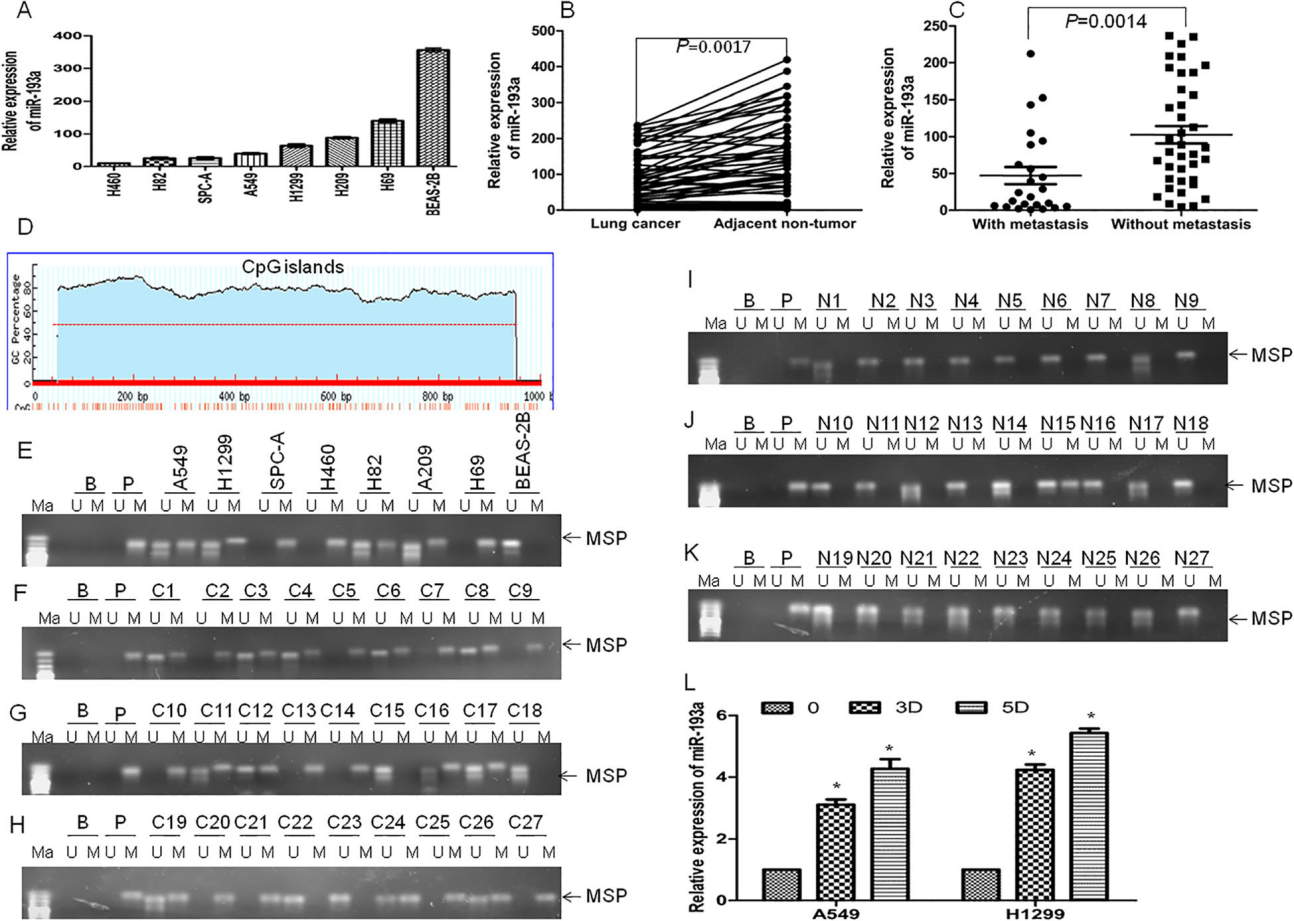
miR-193a was downregulated in lung cancer cells due to DNA hypermethylation. **a** The expression of miR-193a was detected in several lung cancer cell lines and normal lung epithelial cell line BEAS-2B by qRT-PCR. **b** The expression of miR-193a was measured in lung cancer tissues and adjacent non-tumor tissues. *n* = 62. **c** The expression of miR-193a was detected in lung cancer tissues with metastasis (*n* = 24) or without metastasis (*n* = 38). **d** CpG islands around the region encoding pre-miR-193a were analyzed by MethPrimer software. **e** The methylation status of miR-193a was analyzed by MSP in several lung cancer cell lines and BEAS-2B. Ma: DNA marker; B: Blank; P: positive control of methylated DNA. Bands of ‘M’ or ‘U’ are PCR products from methylation-specific or unmethylation-specific primers, respectively. **f**–**k** The methylation status of miR-193a was analyzed by MSP in 27 lung cancer tissues (**f**–**h**) and 27 adjacent non-tumor tissues (**i**–**k**). **l** The expression of miR-193a was detected in A549 and H1299 cells treated with 5 μM AZA for 3 and 5 days. *P < 0.01 versus untreated cells

To investigate the role of miR-193a in lung cancer cells, the expression of miR-193a was detected in several lung cancer cell lines and normal lung epithelial cell line BEAS-2B. The level of miR-193a was significantly reduced in lung cancer cell lines compared with BEAS-2B (Fig. 1a). To confirm the low expression of miR-193a in lung cancer cells, the relative expression of miR-193a was detected in lung cancer tissues and adjacent non-tumor tissues. MiR-193a was downregulated in about 81 % of tumors (*P* < 0.01, 50 of 62 patients), with about 40 % reduction relative to their adjacent non-tumor tissues by qRT-PCR (Fig. 1b). The relationship between miR-193a expression levels and clinicopathological characteristics of the NSCLC patients were indicated in Additional file 1: Table S1. No statistically significant correlations were observed between the miR-193a expressions and age, gender, tumor size, or degree of differentiation, respectively. However, the expression of miR-193a was significantly decreased in lung cancer with advanced stages (TNM III and IV) compared with early stages (Stage I + II, Additional file 1: Table S1). Also, miR-193a expression was reduced in lung cancer patients with metastasis compared with those without metastasis (Fig. 1c and Additional file 1: Table S1).

Gene silencing is often mediated by promoter hypermethylation in different kinds of cancers [28]. Using MethPrimer software [27], we identified typical CpG islands around the region encoding pre-miR-193a, suggesting that miR-193a was likely regulated by DNA methylation (Fig. 1d). To further determine whether the CpG islands of pre-miR-193a were hypermethylated in a tumor-specific manner, the methylation status of miR-193a was analyzed by methylation-specific PCR (MSP) in several lung cancer cell lines and BEAS-2B. Hypermethylation of miR-193a was observed in seven lung cancer cell lines (Fig. 1e). In contrast, a complete absence of miR-193a methylation was found in BEAS-2B cells (Fig. 1e). Furthermore, we investigated the status of methylation in lung cancer tissues and adjacent non-tumor tissues. Similarly, 23 of 27 DNA from lung cancer tissues presented hypermethylation (Fig. 1f–h). However, hypermethylation was only detected in 1 of 27 adjacent non-tumor tissues (Fig. 1i–k). To further explore whether DNA hypermethylation mediates the silencing of miR-193a, A5459 and H1299 cells were treated with DNA methyltransferase inhibitor 5′-azacytidine (AZA) and the levels of miR-193a was measured. AZA increased miR-193a expression in a time-dependent manner (Fig. 1l).

### Ectopic expression of miR-193a inhibits metastasis in lung cancer cells

**Fig. 2:**
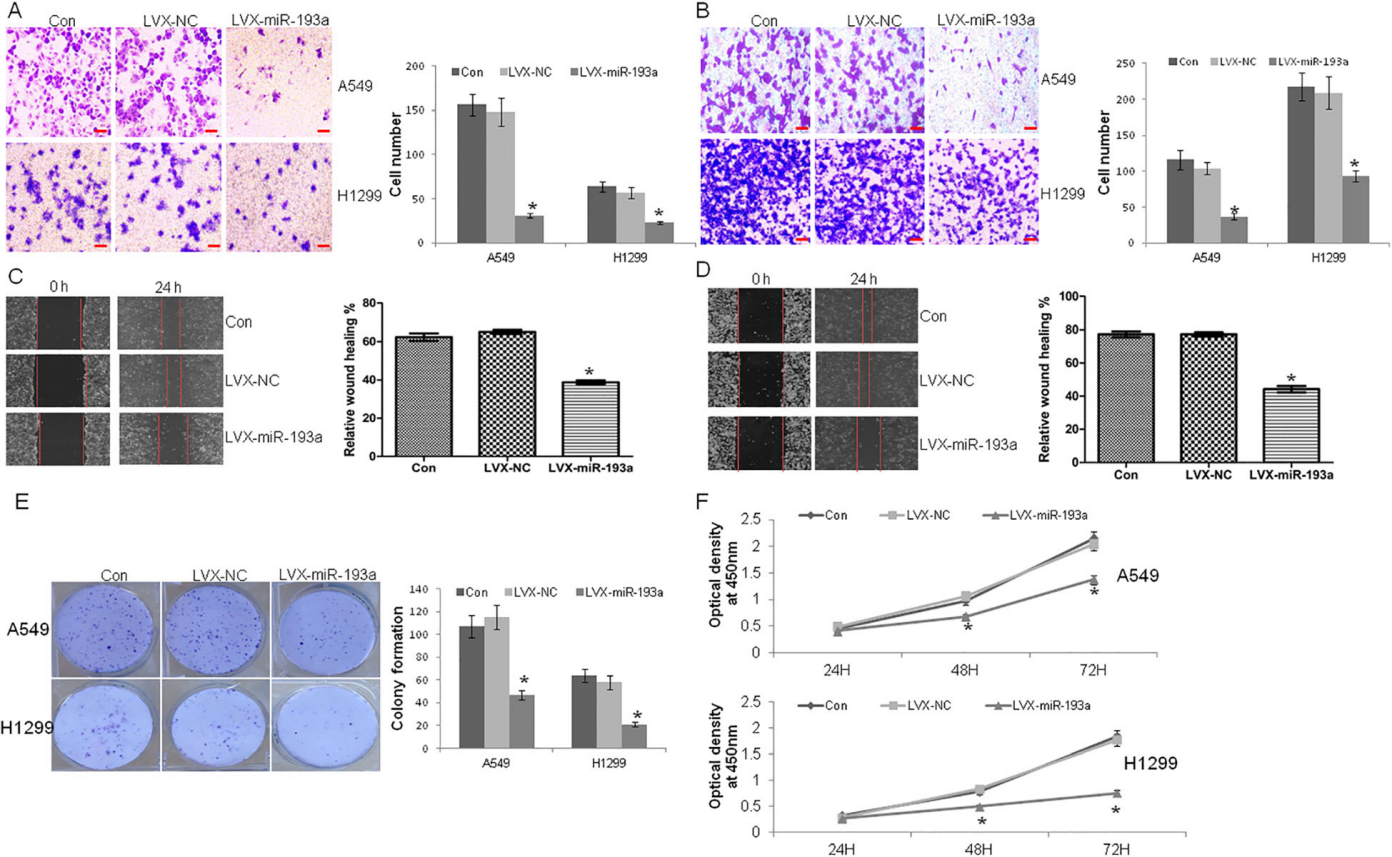
The anti-metastasis activity of miR-193a in lung cancer cells. **a** and **b** Transwell migration (**a**) and invasion (**b**) assays were performed in A549 and H1299 cells transfected with lentivirus LVX-miR-193a or negative control LVX-NC. *P < 0.01 versus NC. **c** and **d** Wound healing assay was measured in A549 (**c**) and H1299 cells (**d**), which were transfected with LVX-miR-193a or LVX-NC. *P < 0.01 versus NC. **e** Colony formation was performed by methylene blue staining in A549 and H1299 cells transfected with LVX-miR-193a or LVX-NC. *P < 0.01 versus NC. **f** Cell proliferation was detected by CCK-8 assay in A549 and H1299 cells transfected with LVX-miR-193a or LVX-NC for 24, 48, and 72 h. Scale bar = 50 μm. The results are representative of at least three independent experiments. Statistical analysis was performed using Student’s *t*-test

The reduced expression of miR-193a prompted us to investigate the biological role of miR-193a in lung cancer. Migration and invasion were evaluated in A549 and H1299 cells transfected with lentivirus vector LVX-NC or LVX-miR-193a. The relative expressions of miR-193a were elevated by 93- and 102-fold in LVX-miR-193a-transfected cells compared with normal control in A549 and H1299 cells (data not shown), respectively. MiR-193a reduced the migration (Fig. 2a) and invasion (Fig. 2b) in A549 and H1299 cells compared with negative control. Meanwhile, overexpression of miR-193a significantly decreased the wound healing activity in A549 (Fig. 2c) and H1299 cells (Fig. 2d). Further, overexpression of miR-193a decreased the colony formation (Fig. 2e) and suppressed cell proliferation (Fig. 2f).

### MiR-193a directly targets WT1 and indirectly modulates E-cadherin

**Fig. 3:**
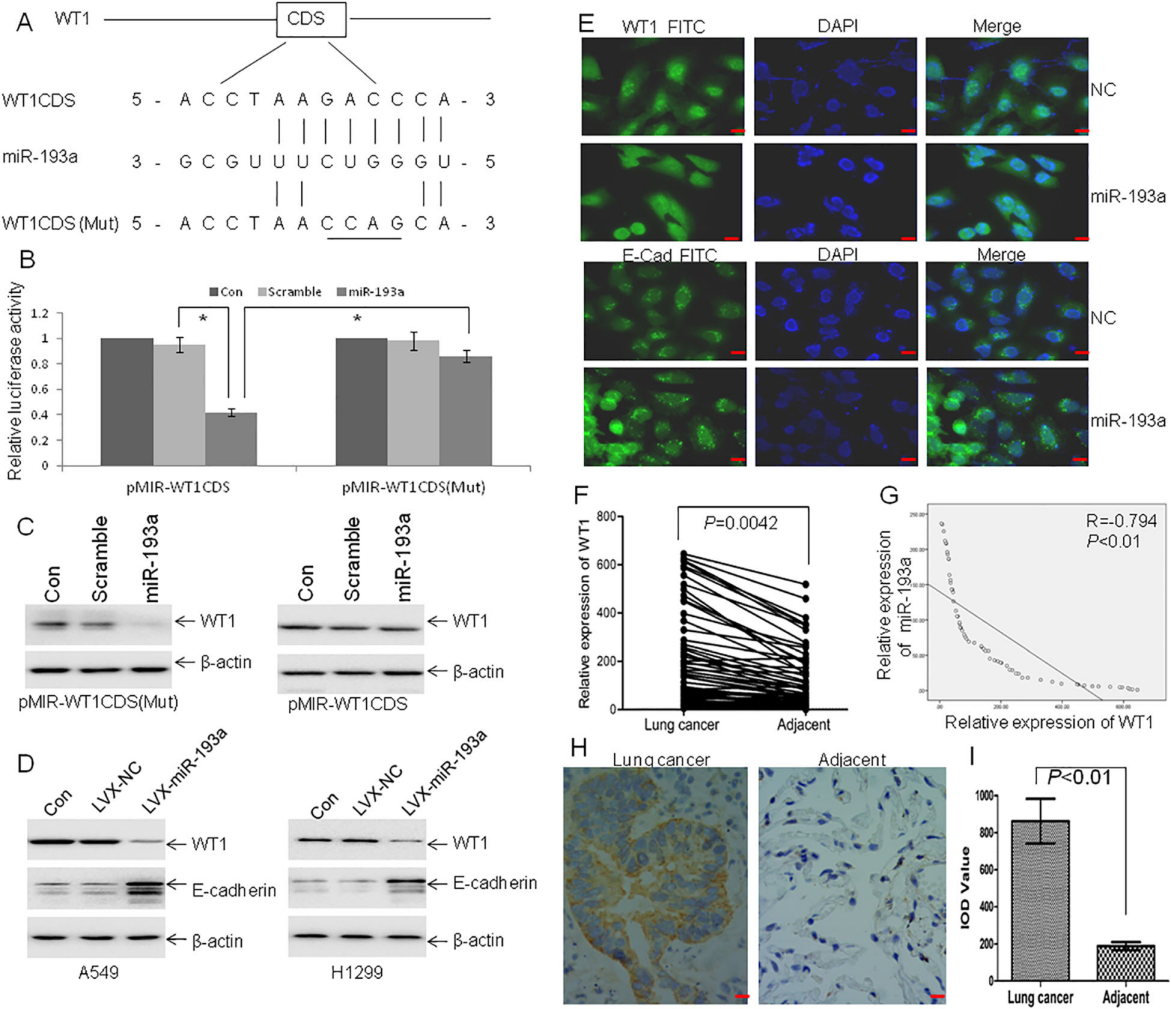
miR-193a modulates E-cadherin expression through targeting WT1. **a** Schematic of putative binding sites for miR-193a in WT1 CDS. **b** A549 cells were transfected with wide-type pMIR-WT1CDS, pMIR-WT1CDS (Mut), pMIR-NC, and the pRL-SV40 containing Renilla luciferase gene for 24 h, followed by the transfection with miR-193a mimic or Scramble for another 24 h. Firefly and Renilla luciferase activities were both detected and histograms showed the Firefly luciferase activities normalized to Renilla luciferase activities. *P < 0.01 versus Scramble. **c** Western blot for WT1 was performed in lysates from A549 cells, which were treated as Fig. 3b. **d** WT1 and E-cadherin expressions were performed in A549 and H1299 cells transfected with lentivirus LVX-miR-193a or LVX-NC. **e** Immunofluorescence (IF) staining assay for WT1 and E-cadherin was analyzed in A549 cells transfected with LVX-miR-193a or LVX-NC. Scale bar = 50 μm. **f** *WT1* expression was measured in lung cancer tissues and adjacent non-tumor tissues by qRT-PCR. *n* = 62. **g** The plotting of *miR-193a* versus *WT1* expression showed an inverse correlation between them. A statistically significant correlation between miR-193a versus WT1 expression was observed by Pearson’s method. *n* = 62. **h** A representative picture of immunohistochemical staining of WT1 in an adenocarcinoma (*left*) and adjacent non-cancer specimen (*right*). **i** Average value of integrated optical density (IOD) was evaluated by analyzing six fields per slide and recorded in the histogram. *n* = 20

To determine potential target genes by miR-193a, several miRNAs predicting software including miRwalk, TargetScan, and PicTar were used to predict possible target genes. MiR-193a has an ability to bind CDS of WT1 (Fig. 3a). CDS of WT1 including putative miR-193a-binding sites was subcloned into pMIR vector to produce wide-type vector pMIR-WT1CDS and mutation vector pMIR-WT1CDS(Mut) (Fig. 3a). These vectors were co-transfected together with miR-193a mimics or its negative control into A549 cells. Overexpression of miR-193a decreased the luciferase activities by approximately 40 %, which were almost abolished by the mutation in putative miR-193a-binding sites (Fig. 3b). Furthermore, miR-193a minics decreased the protein expression of WT1 in cells transfected with pMIR-WT1CDS (Mut) (Fig. 3c, Left). However, miR-193a minics failed to decrease WT1 expression in cells transfected with pMIR-WT1CDS (Fig. 3c, Right). We speculated that pMIR-WT1CDS binds miR-193a minics and consequently prevents miR-193a-induced decrease of WT1. To further explore the role of miR-193a in lung cancer cells, A549 and H1299 cells were transfected with lentivirus vector LVX-miR-193a or negative control. Overexpression of miR-193a significantly decreased the protein levels of WT1, but increased the expression of the epithelial marker E-cadherin (Fig. 3d), which was reported to be negatively regulated by WT1 in NSCLC [15]. Immunofluorescent analysis also showed that the expression of WT1 was decreased but E-cadherin was increased in miR-193a-transfected cells compared with negative control (Fig. 3e and Additional file 3: Figure S1A).

Because previous data showed that WT1 was increased in hematological malignancies and multiple types of cancers [12, 29], we then determined whether the expression of WT1 was increased in lung cancer tissues. We investigated 62 pairs of NSCLC (Additional file 1: Table S1) and corresponding adjacent specimens using qRT-PCR. Consistent with previous report [30], the level of *WT1* in lung cancer tissues was higher than that in paired adjacent non-cancer ones (Fig. 3f). We further analyzed the relationship between *WT1* and *miR-193a* expressions. Importantly, a significant inverse correlation between *miR-193a* and *WT1* mRNA expressions was found in lung cancer tissues (Fig. 3g). Finally, we confirmed the overexpression of WT1 by IHC in 20 lung cancer tissues (11 adenocarcinoma and 9 squamous cell carcinomas). The average value of integrated optical density (IOD) was higher in lung cancer tissues compared with paired adjacent non-cancer ones (Fig. 3h and i).

### Knockdown of WT1 inhibits metastasis

**Fig. 4:**
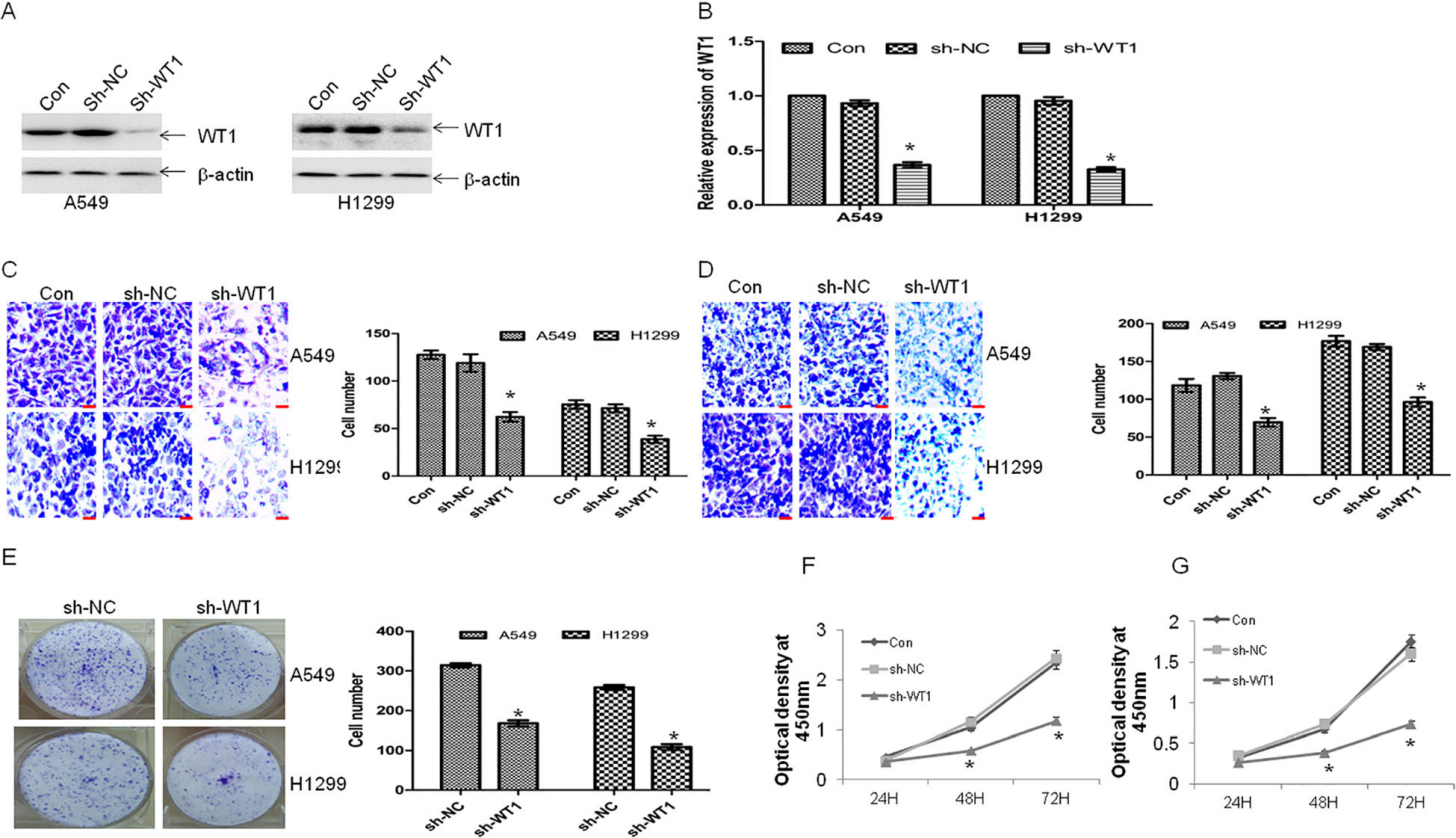
Knockdown of WT1 resembles the anti-metastasis activity by miR-193a. A549 and H1299 cells were transfected with specific shRNA for WT1 or nonspecific shRNA (sh-NC). **a** and **b** WT1 protein and mRNA levels were detected in A549 and H1299 cells transfected with sh-NC or sh-WT1. *P < 0.01 versus sh-NC. **c** and **d** Transwell migration (**c**) and invasion (**d**) assays were performed in A549 and H1299 cells transfected with sh-NC or sh-WT1. Scale bar = 50 μm. *P < 0.01 versus sh-NC. **e** Colony formation was performed by methylene blue staining in A549 and H1299 cells transfected with sh-NC or sh-WT1. *P < 0.01 versus sh-NC. **f** and **g** Cell proliferation was detected by CCK-8 assay in A549 (**f**) and H1299 (**g**) cells transfected with sh-NC or sh-WT1 for 24, 48, and 72 h. *P < 0.01 versus sh-NC

Having shown that miR-193a negatively regulates WT1 expression and inhibits metastasis, we directly address the role of WT1 downregulation in the anti-metastasis activity. To determine whether WT1 knockdown resembles the effects of miR-193a in our experimental model, lung cancer cells were transfected with specific shRNA for WT1 (sh-WT1). As shown in Fig. 4a and b, the mRNA and protein levels of WT1 were decreased by sh-WT1. Accordingly, knockdown of WT1 reduced the migration (Fig. 4c) and invasion (Fig. 4d) in lung cancer cells. Furthermore, WT1 knockdown reduced colony formation (Fig. 4e) and decreased cell growth in A549 (Fig. 4f) and H1299 cells (Fig. 4g).

### Ectopic overexpression of WT1 partially reverses miR-193a-induced inhibition of metastasis

**Fig. 5:**
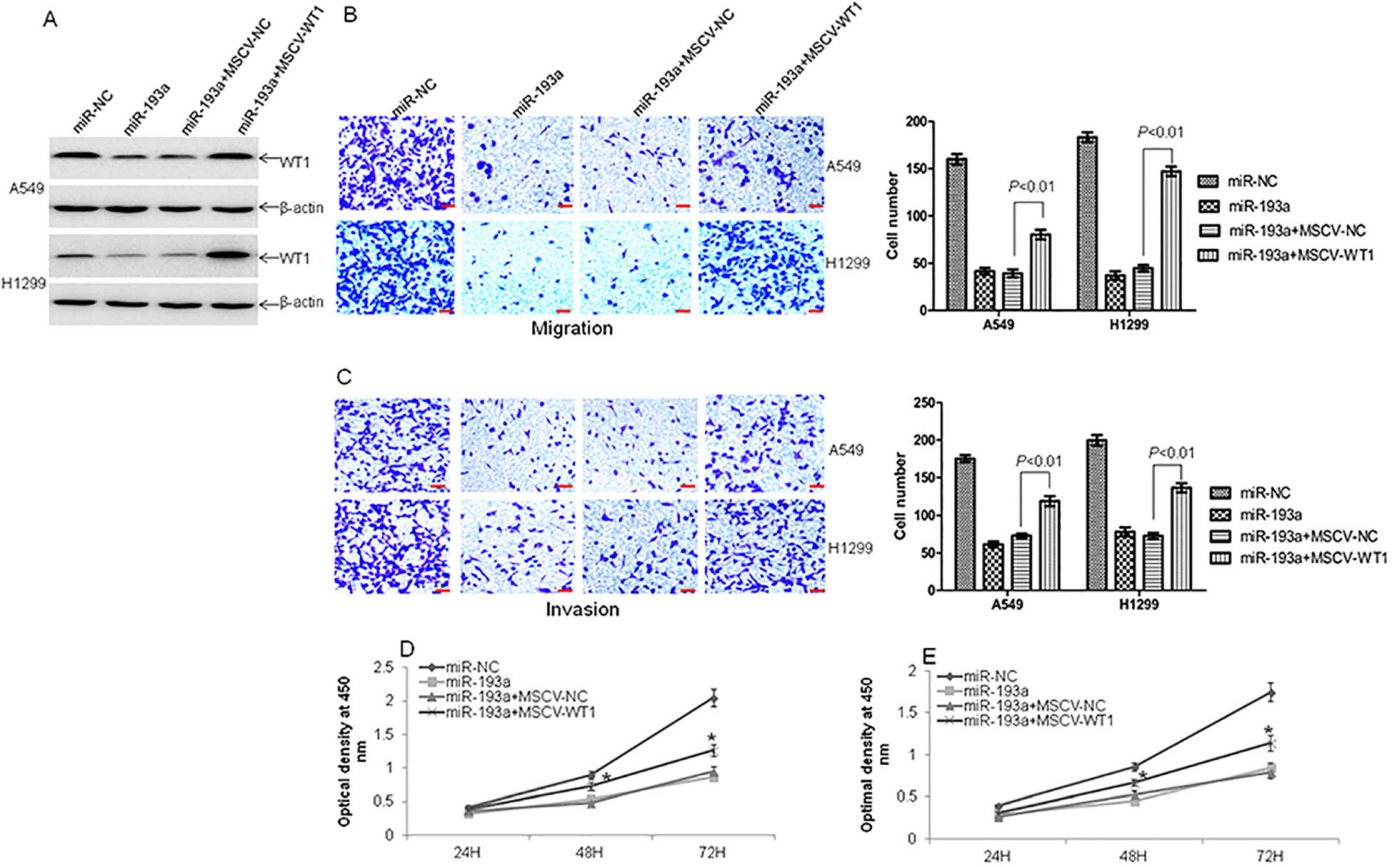
miR-193a-induced inhibition of metastasis is partly prevented by ectopic overexpression of WT1. **a** Western blot was analyzed in A549 and H1299 cells transfected with LVX-miR-193a and retrovirus MSCV-NC or MSCV-WT1. **b** and **c** Transwell migration (**b**) and invasion (**c**) assays were performed in these cells. **d** and **e** Cell proliferation was detected by CCK-8 assay in A549 (**d**) and H1299 (**e**) cells, which were transfected with LVX-miR-193a and retrovirus MSCV-NC or MSCV-WT1 for 24, 48, and 72 h. *P < 0.01 compared with MSCV-NC

Because miR-193a inhibited metastasis via targeting WT1 protein, which acts as an oncogene in multiple types of cancers including lung cancer, we are interested in examining whether WT1 counteracts miR-193a-induced anti-metastasis. Migration and invasion were measured in miR-193a-overexpressed A549 and H1299 cells, which were transfected with MSCV-NC or MSCV-WT1. As shown in Fig. 5a, miR-193a-induced inhibition of WT1 was prevented by transfection of MSCV-WT1 compared with negative control. Correspondingly, overexpression of WT1 partially reversed the anti-migration (Fig. 5b) and anti-invasion (Fig. 5c) induced by miR-193a. Further, overexpression of WT1 partially prevented miR-193a-induced growth inhibition (Fig. 5d and e). Finally, migration and invasion were measured in miR-193a-overexpressed A549 cells transfected with MSCV-NC or MSCV-WT1 CDS (Mut). As shown in Additional file 4: Figure S2A-C, overexpression of WT1 CDS (Mut) did not prevent the anti-migration and anti-invasion induced by miR-193a.

### TGF-β1-induced EMT is attenuated by the overexpression of miR-193a

**Fig. 6:**
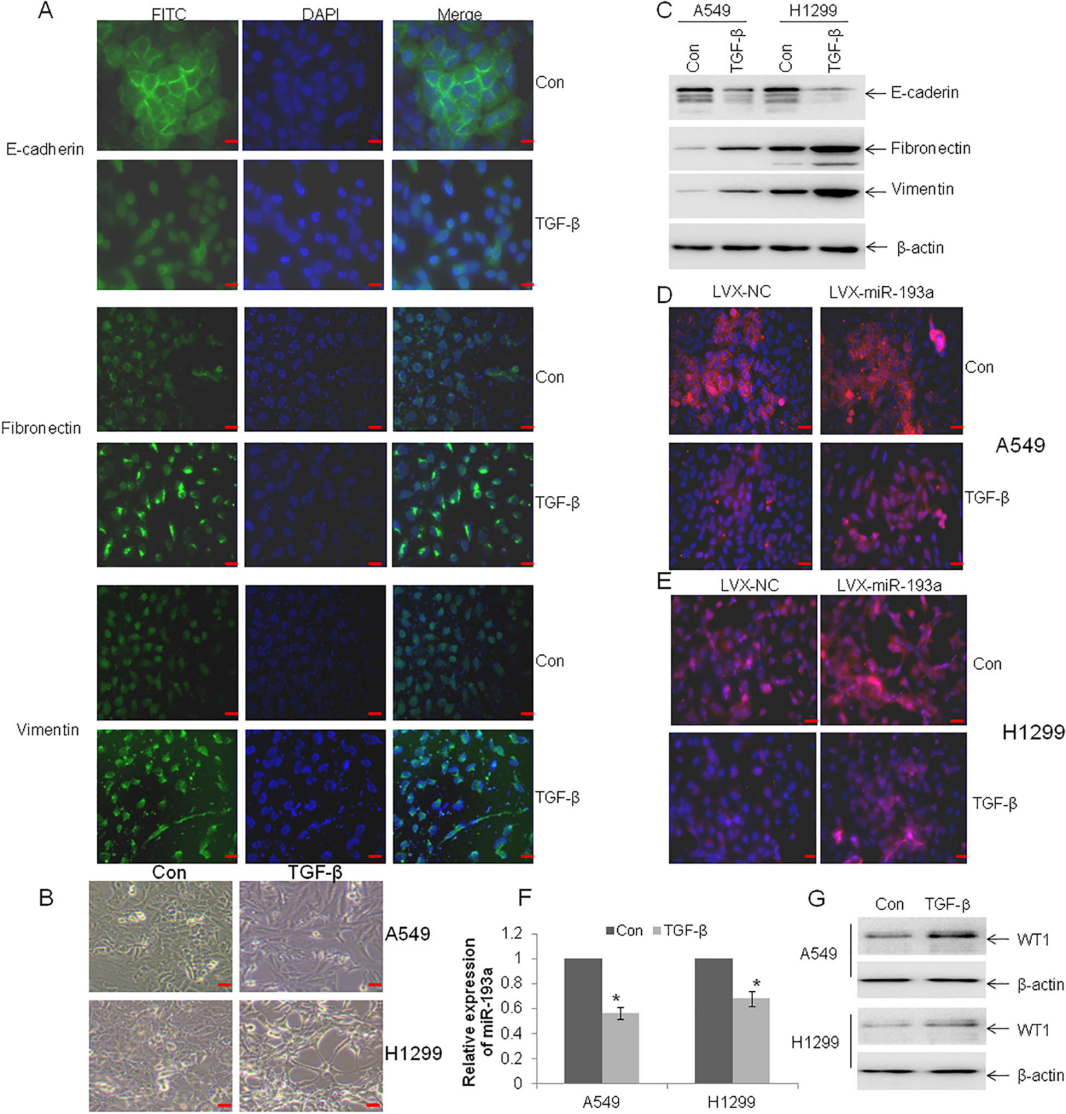
Overexpression of miR-193a partly reverses TGF-β1-induced EMT. A549 and H1299 cells were treated with 10 ng/ml TGF-β1 for 3 days. **a** Immunofluorescence staining was performed for epithelial marker E-cadherin and the mesenchymal markers fibronectin and vimentin in A549 cells. **b** Morphologic changes associated with EMT at 3 days were shown in the phase contrast images. **c** Western blot for E-cadherin, fibronectin, and vimentin in A549 and H1299 cells treated with TGF-β1 for 3 days. **d** and **e** A549 and H1299 cells were transfected with LVX-NC or LVX-miR-193a, followed with treatment with TGF-β1 for 3 days. Immunofluorescence staining assay was performed for E-cadherin in these cells. **f** and **g** The expressions of miR-193a (**f**) and WT1 (**g**) were measured in A549 and H1299 cells treated with TGF-β1 for 3 days by qRT-PCR and western blot, respectively

The observation that overexpression of miR-193a inhibited metastasis through WT1-E-cadherin axis urged us to investigate whether miR-193a is mediated in the EMT of lung cancer cells. TGF-β1 promotes tumor progression through enhancing migration, invasion, and proliferation, in part by its ability to induce EMT [31]. Decreased expression of the epithelial marker E-cadherin and increased expression of the mesenchymal markers fibronectin and vimentin were found in A549 and H1299 cells treated with 10 ng/ml TGF-β1 for 3 days by immunofluorescence (Fig. 6a and Additional file 5: Figure S3A), morphology (Fig. 6b), and western blot (Fig. 6c). A549 and H1299 cells displayed a spindle-shape and fibroblast-like morphology in the presence of TGF-β1 compared with a classic epithelial morphology in the absence of TGF-β1 (Fig. 6b). All these data suggest that TGF-β1 could successfully induce EMT in A549 and H1299 cells. To further determine the role of miR-193a in the TGF-β1-induced EMT, A549 and H1299 cells transfected with LVX-miR-193a or negative control were treated with TGF-β1 for 3 days. TGF-β1-induced EMT was partially prevented by the overexpression of miR-193a (Fig. 6d and e). Because miR-193a expression was more decreased in lung cancer cells with greater migration and invasion abilities, we speculated that TGF-β1 inhibited the expression of miR-193a. As expected, TGF-β1 reduced the expression of miR-193a by 30 %–50 % (Fig. 6f) and subsequently increased the expression of WT1 in A549 and H1299 cells (Fig. 6g).

### MiR-193a reduces tumorigenicity in a xenograft mouse model

**Fig. 7:**
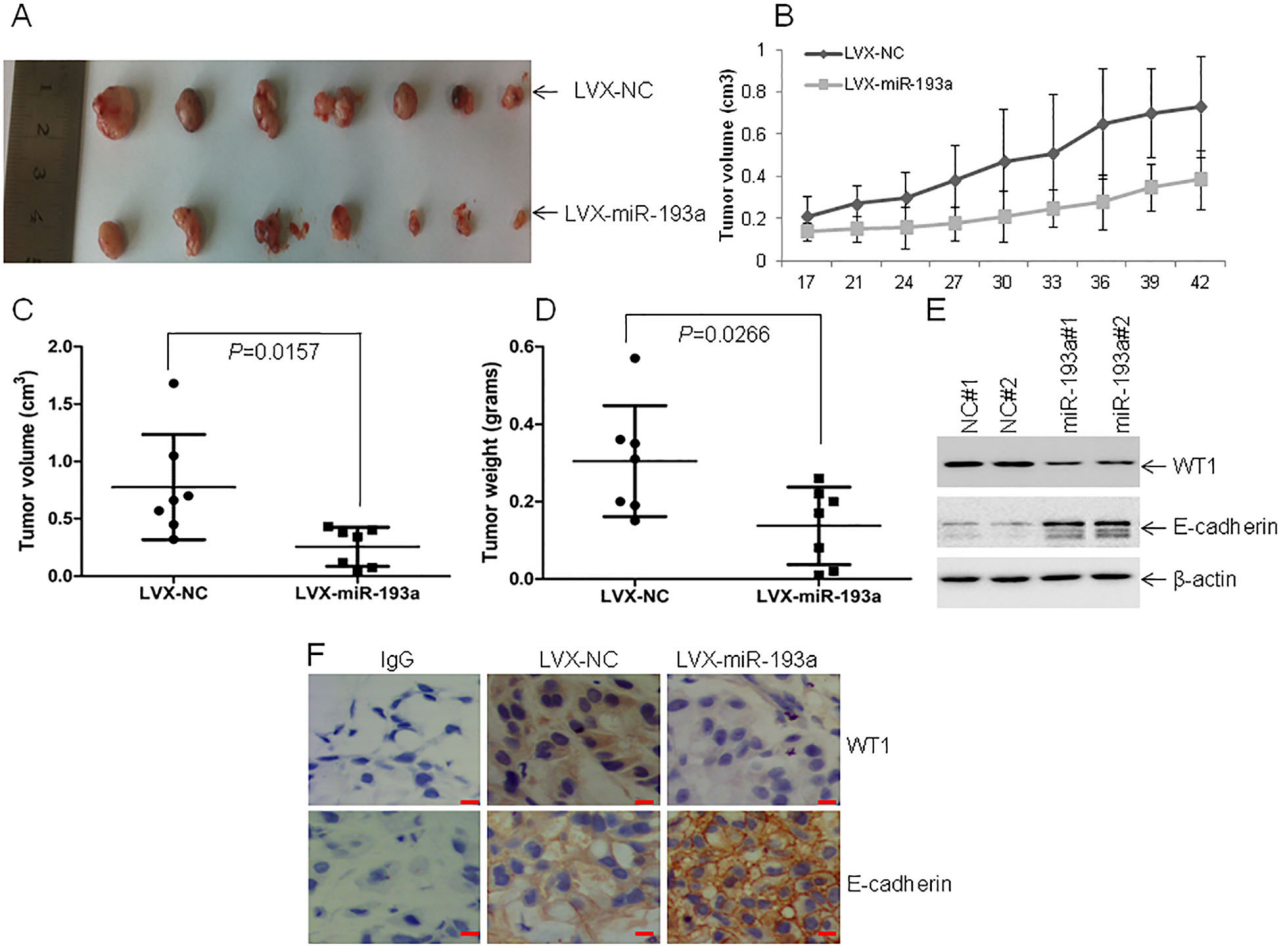
The anti-tumor effects of miR-193a in tumor xenograft. About 1 × 10^7^ viable A549 cells transfected with pLVX-miR-193a (A549-miR-193a) or pLVX-miR-NC (A549-miR-NC) were subcutaneously injected into right flank of each nude mouse. *n* = 7. **a** A photograph of tumors from mice inoculated with A549-miR-193a or A549-miR-NC. **b** Volumes of all tumors were detected every 3 days after 2 weeks. **c** Volumes of all tumors were measured when the experiment was terminated at six weeks after tumor cell inoculation. **d** Net weights of all tumors were measured at the termination of the experiment. **e** The protein levels of WT1 and E-cadherin were detected in two tumor lysates from mice inoculated with A549-miR-193a or A549-miR-NC. **f** IHC staining of WT1 and E-cadherin was done in tumor xenografts from mice inoculated with A549-miR-193a or A549-miR-NC cells. Scale bar = 50 μm

Finally, we determined whether overexpression of miR-193a could reduce the tumorigenicity in a xenograft model. Tumors in mice inoculated with LVX-miR-193a were significantly smaller than those in control mice (Fig. 7a). Furthermore, tumor growth was significantly reduced in mice inoculated with LVX-miR-193a (Fig. 7b). Similarly, the average tumor volume and average tumor weight in mice inoculated with LVX-miR-193a was reduced by 47.2 % (Fig. 7c) and 54.9 % (Fig. 7d) compared with negative control, respectively. Consistent with the results from cell lines, the protein levels of WT1 were significantly decreased but E-cadherin expressions were increased in two tumors obtained from miR-193a-overexpressed mice in comparison with control mice (Fig. 7e). Finally, IHC staining indicated that the expression of WT1 was decreased while E-cadherin was increased in tumor xenograft from mice inoculated with A549-miR-193a than that inoculated with A549-miR-NC (Fig. 7f).

## Discussion

MiRNAs have critical roles in carcinogenesis and metastasis. Identification of tumor-related miRNAs and their direct target genes is important for understanding the biological significance of miRNAs in tumorigenesis. In this study, we investigated an anti-metastasis role of miR-193a in NSCLC. Our results indicate that decreased expression of miR-193a contributes to proliferation, metastasis, and TGF-β-induced EMT through WT1-E-cadherin pathway. Consistently, ectopic overexpression of miR-193a inhibits metastasis and the EMT-like conversion in NSCLC. Intriguingly, miR-193a expression is decreased in TGF-β-induced EMT, suggesting that miR-193a-WT1-E-cadherin pathway was mediated in TGF-β-induced EMT. Therefore, our results suggest that miR-193a exerts tumor-suppressive effects through WT1-E-cadherin pathway.

Dysregulation of miRNAs is frequently observed in various kinds of cancer and miRNAs can function as oncogenes or tumor suppressive genes. In our study, the levels of miR-193a were lower in lung cancer tissues than those in normal noncancerous tissues, suggesting that miR-193a might be involved in the pathogenesis of lung cancer as a tumor suppressor. Further, lower expression of miR-193a was related with greater migration and invasion. Indeed, many studies have indicated that miR-193a was down-regulated in various types of cancers [32–34]. Consistently, miR-193a-3p, the another product of pre-miR-193a, was also downregulated in lung cancer [25, 34]. DNA methylation has been suggested as one of the mechanisms which might be associated with miRNA silencing. Our studies also indicate that hypermethylation of miR-193a is frequently occurred in lung cancer cell lines and lung cancer tissues but not in adjacent non-tumor tissues. These observations are compatible with the work by Heller et al. that miR-193a was silenced by DNA hypermethylation through genome-wide miRNA expression profiling [35].

TGF-β induces the repression of E-cadherin through several well recognized transcriptional repressors, such as Snail, Slug, Twist, Zeb 1/2, and E47 [36]. Here, we report that miR-193a-mediated pathway may take part in TGF-β-induced inhibition of E-cadherin (Additional file 6: Figure S4A). The further decrease of miR-193a by TGF-β loses the ability of suppressing WT1 and finally enhances the expression of WT1, which leads to the decreased expression of E-cadherin (Additional file 6: Figure S4B). Several reports also indicated that miRNAs modulated EMT through different mechanisms [37]. For example, TGF-β-induced inhibition of miR-200 led to the increased expression of Zeb, which finally decreased the expression of E-cadherin in breast cancer cells [38, 39]. TGF-β increased the expression of miR-181a to promote EMT-like change in cirrhosis and hepatocellular cancer [40]. Therefore, TGF-β-miR-193a-E-cadherin pathway complements the routine regulatory net in EMT. However, further studies are needed to determine the underlying mechanism by which miR-193a is decreased by TGF-β.

In most normal cells, E-cadherin binds β-catenin to maintain normal cell structure. This complex forms a powerful molecular barrier that prevents the proliferation, invasion, and metastasis. During EMT process, the loss of E-cadherin leads to the damaged junctions and decreased contact inhibition. Consequently, β-catenin enters the nucleus and activates Wnt/β-catenin signaling to induce the expression of transcript factors such as Snails and Zebs, which finally inhibit the expression of E-cadherin encoding gene *CDH1* [41]. Therefore, the loss of E-cadherin expression is considered as the important step for EMT process. In most fully differentiated cancer tissues, E-cadherin is highly expressed and predicts better outcome. However, some reports indicate that many invasive and metastatic cancers maintain high expression of E-cadherin [42]. For example, overexpression of E-cadherin in human breast cancer cell line MDA-MB-435 does not inhibit motility and metastasis [43]. Another study indicates that E-cadherin expression is similar in breast ductal carcinoma and in normal glands [42]. High expression of E-cadherin is discovered in malignant ovarian carcinomas and promotes the malignant transformation of ovarian epithelial cells [44]. Similarly, high expression of E-caderin is positively correlated with the invasive growth and infiltration of prostate cancer cells [45]. These reports indicate that E-cadherin expression is not associated with metastatic status or mesenchymal phenotype. Also, this observations indicate that E-cadherin don’t prevent the expansion of carcinoma cells into surrounding tissues. This discrepancy might be due to the complex role of E-cadherin in metastasis or the different responses of cells which are treated with stimuli. However, most studies indicate that the reduction of E-cadherin expression in lung cancer cells is associated with low tumor differentiation, high metastasis, and poor outcome [46, 47]. Our data affirm these results and indicate a new TGF-β-miR-193a-E-cadherin pathway to promote TGF-β-induced EMT in lung cancer.

MiRNAs have been implied to play crucial roles of EMT in NSCLC. In addition to miR-193a, several other miRNAs are mediated in the EMT of NSCLC. For example, miR-149 expression was downregulated in lung cancer and was inversely correlated with invasive capability in NSCLC. Furthermore, miR-149 inhibited EMT via directly targeting Forkhead box M1 (FOXM1), which was involved in the TGF-β-induced EMT [48]. The expression of miR-132 was significantly decreased in NSCLC cell lines and clinical NSCLC cancer tissues. Mechanistically, overexpression of miR-132 suppressed EMT process via inhibiting ZEB2-E-cadherin signaling [49]. MiR-134/487b/655 cluster, which was increased by TGF-β, contributed to TGF-β-induced EMT through the loss of PTEN stability [50]. Our finding that miR-193a inhibits TGF-β-induced EMT through WT1-E-Cadherin axis augments the role of miRNAs in EMT of NSCLC. Therefore, completely investigating the whole miRNAs mediated in the process of EMT might facilitate the knowledge of pathogenesis in NSCLC.

A single miRNA can target multiple genes, whereas multiple miRNAs can target a single gene. Thus, miR-193a might have multiple different mRNA targets other than WT1, and these additional targets may also play important roles in carcinogenesis. In addition to *WT1*, miR-193a targeted several important oncogene, such as *Yin Yang1* (YY1) [33], *phosphoinositide-3-kinase regulatory subunit* (*3PIK3R3*), and *the mammalian target of rapamycin* (*mTOR*) [34]. Similarly, besides miR-193a, miR-125a was identified to suppress WT1 expression via binding 3′UTR of WT1 in myeloid leukemia cells [51]. Our previous data also indicated that miR-15a/16-1 inhibited the expression of WT1 probably through an indirect mechanism in leukemic cells [52]. Thus, elucidating the complex regulatory net of WT1 by miRNAs may shed light on the high expression of WT1 in cancer cells.

Most of studies demonstrate that WT1 is overexpressed in lung cancer cells and WT1 acts as oncogene to promote proliferation and metastasis [12, 30]. For example, Xu et al. reported that WT1 facilitated NSCLC cell proliferation by up-regulating Cyclin D1 and p-pRb expressions [12]. Wang et al. demonstrated that isoform C of WT1 was overexpressed in lung cancer and WT1 facilitated the carcinogenesis of lung cancer via regulating PI3K/AKT signaling pathway [53]. However, whether the higher expression of WT1 predicted poor metastasis in lung cancer remains controversial. Hayashi S et al. reported that low expression of WT1 was a negative prognostic factor in NSCLC tumors and was also associated with lymph node metastasis [54]. In addition, Moriya and his colleagues indicated that high level of WT1 was associated with the suppression of lymph node metastasis in patients with human lung squamous cell carcinoma (SCC) [55]. The possible reason for this discrepancy is likely due to the complex isoforms of WT1 [14]. At least eight major isoforms of WT1 were isolated from a cDNA library in lung cancer tissues [55]. However, the role of single WT1 isoform in lung cancer is large unknown. Further studies are thus needed to resolve this discrepancy.

Inhibition of WT1 in solid cancers and hematological malignancies demonstrates potential anti-tumor activities. WT1 knockdown by antisense oligomers [56], ribozyme [57], or PEI- RNAi [58] suppresses cell proliferation and induces apoptosis, suggesting that removal of WT1 might has anti-cancer effect in multiple types of cancers. Additionally, WT1 peptide-based immunotherapy shows good clinical responses, such as reduction in tumor sizes, demonstrating that WT1 vaccination should be a promising treatment for patients with lung cancer, breast cancer, and leukemia [59]. Therefore, inhibiting or degrading WT1 protein might contribute to the clinical treatment for WT1-overexpressed lung cancer patients.

## Conclusions

In summary, we explore the role of miR-193a-WT1-E-cadherin axis in the metastasis and EMT in NSCLC. MiR-193a expression is decreased due to DNA hypermethylation in NSCLC specimens. Restoration of miR-193a inhibits migration, invasion, and TGF-β1-induced EMT through modulating WT1-E-cadherin axis. Furthermore, overexpression of WT1 partially prevents miR-193a-induced inhibition of migration and invasion, suggesting that WT1 plays an important role in the anti-metastasis by miR-193a. Thus, restoring the expression of miR-193a or decreasing the expression of WT1 might provide alternative therapeutic strategy for NSCLC patients.

## Additional files

Additional file 1: Table S1. Relationship between the expression of miR-193a and clinicopathologic parameters. (DOCX 16 kb)
Additional file 2: Table S2. The sequences of primers for qRT-PCR and construction of plasmids. (DOCX 16 kb)
Additional file 3: Figure S1. (A) IF staining for WT1 and E-cadherin was analyzed in H1299 cells transfected with LVX-miR-193a or LVX-NC. (TIF 4152 kb)
Additional file 4: Figure S2. Ectopic overexpression of WT1 CDS (Mut) fails to prevent miR-193a-induced anti-metastasis activity. (A–C) Transwell migration and invasion assays were performed in A549 cells, which were transfected with LVX-miR-193a and MSCV-NC or MSCV-WT1 CDS (Mut). (TIF 4889 kb)
Additional file 5: Figure S3. (A) IF staining was performed for E-cadherin, fibronectin, and vimentin in H1299 cells treated with 10 ng/ml TGF-β1 for 3 days. (TIF 3869 kb)
Additional file 6: Figure S4. (A) A schematic representation of TGF-β1-induced inhibition of E-cadherin through miR-193a-WT1 axis. (B) A schematic demonstration of DNA hypermethylation of miR-193a in lung cancer cells. Hypermethylation of miR-193a leads to the low expression of miR-193a, which loses the ability to inhibit WT1 expression. High expression of WT1 contributes to EMT through decreasing the expression of E-cadherin. (TIF 13002 kb)
