# Erratum to: Pathologically decreased expression of miR-193a contributes to metastasis by targeting WT1-E-cadherin axis in non-small cell lung cancers

**DOI:** 10.1186/s13046-017-0501-9

**Published:** 2017-02-16

**Authors:** Junjie Chen, Shenmeng Gao, Chunjing Wang, Zhonggai Wang, Huxiang Zhang, Kate Huang, Bin Zhou, Haiying Li, Zhijie Yu, Jianbo Wu, Chengshui Chen

**Affiliations:** 1 Department of Respiration, The First Affiliated Hospital of Wenzhou Medical University, Nanbaixiang, Ouhai District, Wenzhou, 325000 Zhejiang Province China; 2 Laboratory of Internal Medicine, The First Affiliated Hospital of Wenzhou Medical University, Nanbaixiang, Ouhai District, Wenzhou, Zhejiang Province China; 3 School of Laboratory Medicine & School of Life Science, Wenzhou Medical University, Nanbaixiang, Ouhai District, Wenzhou, Zhejiang Province China; 4 Pathology Department, The First Affiliated Hospital of Wenzhou Medical University, Nanbaixiang, Ouhai District, Wenzhou, Zhejiang Province China

## Erratum

During production, Chengshui Chen was omitted from the list of corresponding authors in the original article [1]. Both Jianbo Wu and Chengshui Chen are corresponding authors for this article.
