# Supplementary material for: Pathologically decreased expression of miR-193a contributes to metastasis by targeting WT1-E-cadherin axis in non-small cell lung cancers
Source: J Exp Clin Cancer Res. 2016 Nov 7;35:173. doi: 10.1186/s13046-016-0450-8 (PMC5100283; doi:10.1186/s13046-016-0450-8)
Supplement: Additional file 1: Table S1. — Relationship between the expression of miR-193a and clinicopathologic parameters. (DOCX 16 kb) [file 13046_2016_450_MOESM1_ESM.docx]

| Clinicopathologic parameters | Number of cases(62) | Median expression of miR-193a |
| --- | --- | --- |
|  |  | Mean±S.D. *P*-value |
| Age (years)  <60  ≥60 | 23  39 | 78.56±68.91 0.58  82.74±72.45 |
| Gender  Male  Female | 40  22 | 83.57±74.79 0.64  77.53±70.52 |
| Pathological subtypes  [Adenocarcinoma](http://dict.youdao.com/w/adenocarcinoma/#keyfrom=E2Ctranslation)  Squamous cell carcinomas  Others | 29  26  7 | 0.78  81.71±69.15  82.65±72.56  80.65±73.39 |
| Tumor size (cm)  ≤3  >3 | 33  29 | 80.36±72.03 0.43  82.25±74.37 |
| Degree of differentiation  Well and moderately  Poorly | 36  26 | 83.73±67.43 0.37  76.01±69.95 |
| TNM stage  Stage I+II  Stage III+IV | 32  30 | 113.65±96.53 0.019  54.67±45.89 |
| Metastasis  No  Yes | 34  28 | 109.27±91.26 0.0014  51.54±43.28 |

Additional file 1: Table S1: Relationship between the expression of miR-193a and clinicopathologic parameters
