# Supplementary material for: Pathologically decreased expression of miR-193a contributes to metastasis by targeting WT1-E-cadherin axis in non-small cell lung cancers
Source: J Exp Clin Cancer Res. 2016 Nov 7;35:173. doi: 10.1186/s13046-016-0450-8 (PMC5100283; doi:10.1186/s13046-016-0450-8)
Supplement: Additional file 2: Table S2. — The sequences of primers for qRT-PCR and construction of plasmids. (DOCX 16 kb) [file 13046_2016_450_MOESM2_ESM.docx]

| WT1-L | 5′-CAA TCA GGG TTA CAG CAC GG-3′ |
| --- | --- |
| WT1-R | 5′-GCT TGA ATG AGT GGT TGG GG-3′ |
| Actin-L | 5′-TGG CAT CCA CGA AAC TAC CT-3′ |
| Actin-R | 5′-CGT ACA GGT CTT TGC GGA TG-3′ |
| LVX-miR-193a-L | 5′-GGA ATT CCG AGC GTC GTG TAA CCC TTG-3′ |
| LVX-miR-193a-R | 5′-GAC TAG TCG AGC GCA CCT CAC CAC TC-3′ |
| pMIR-WT1CDS-L | 5′-CCC AAG CTT CCC ACT TAC AGA TGC ACA GC-3′ |
| pMIR-WT1CDS-R | 5′-AGC TTT GTT TAA ACT TTC TGA CAA CTT GGC CAC C-3′ |
| MSCV-WT1-L | 5′-GAA GAT CTC TGC AGG ACC CGG CTT CCA C-3′ |
| MSCV-WT1-R | 5′-CCG GAA TTC TCA AAG CGC CAG CTG GAG TT-3′ |
| Sh-WT1 | 5′-GCA GTG ACA ATT TAT ACC AAA-3′ |
| miR-193aMMSP-L | 5′-GTC GAG TTG AGC GTA GGT AAT C-3′ |
| miR-193aMMSP-R | 5′-CGA ATA AAA CGC AAA AAT TAT ACG-3′ |
| miR-193aUMSP-L | 5′-GTT GAG TTG AGT GTA GGT AAT TGA-3′ |
| miR-193aUMSP-R | 5′-CCC AAA TAAA ACA CAA AAA TTA TAC AC-3′ |

**Additional file 2: Table S2: The sequences of primers for qRT-PCR and construction of plasmids**
