# Supplementary figures and images for: Pathologically decreased expression of miR-193a contributes to metastasis by targeting WT1-E-cadherin axis in non-small cell lung cancers
Source: J Exp Clin Cancer Res. 2016 Nov 7;35:173. doi: 10.1186/s13046-016-0450-8 (PMC5100283; doi:10.1186/s13046-016-0450-8)

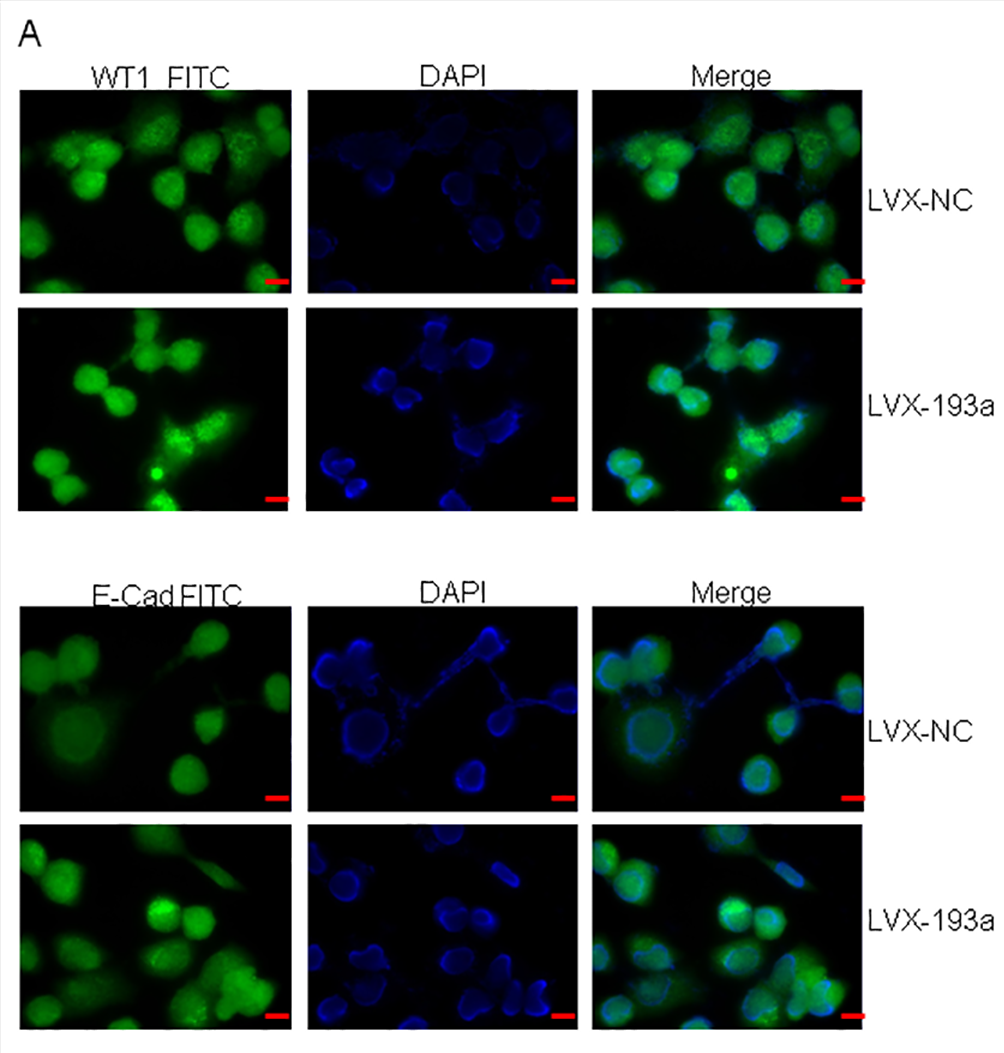

Supplement: Additional file 3: Figure S1. — (A) IF staining for WT1 and E-cadherin was analyzed in H1299 cells transfected with LVX-miR-193a or LVX-NC. (TIF 4152 kb) [file 13046_2016_450_MOESM3_ESM.tif]

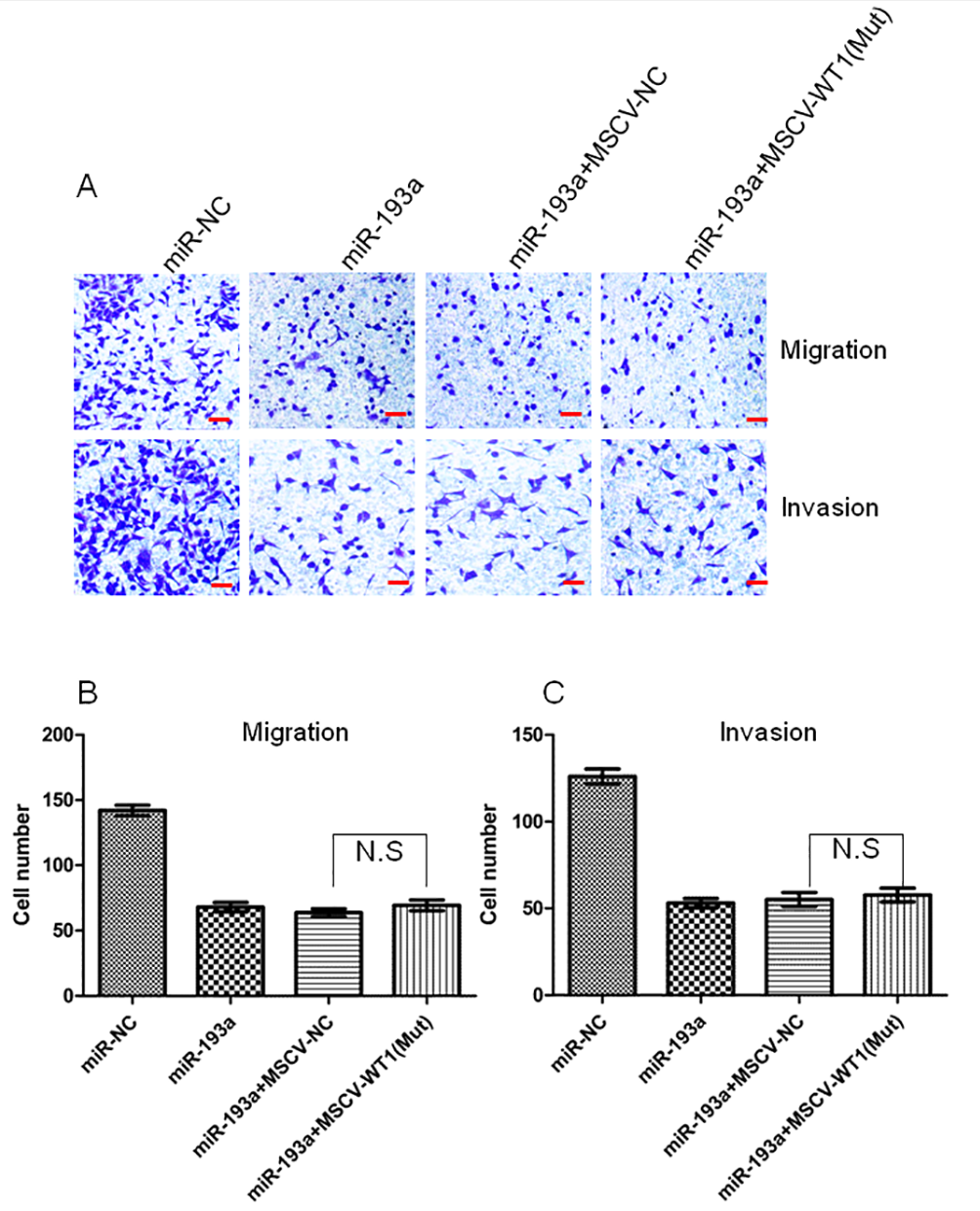

Supplement: Additional file 4: Figure S2. — Ectopic overexpression of WT1 CDS (Mut) fails to prevent miR-193a-induced anti-metastasis activity. (A–C) Transwell migration and invasion assays were performed in A549 cells, which were transfected with LVX-miR-193a and MSCV-NC or MSCV-WT1 CDS (Mut). (TIF 4889 kb) [file 13046_2016_450_MOESM4_ESM.tif]

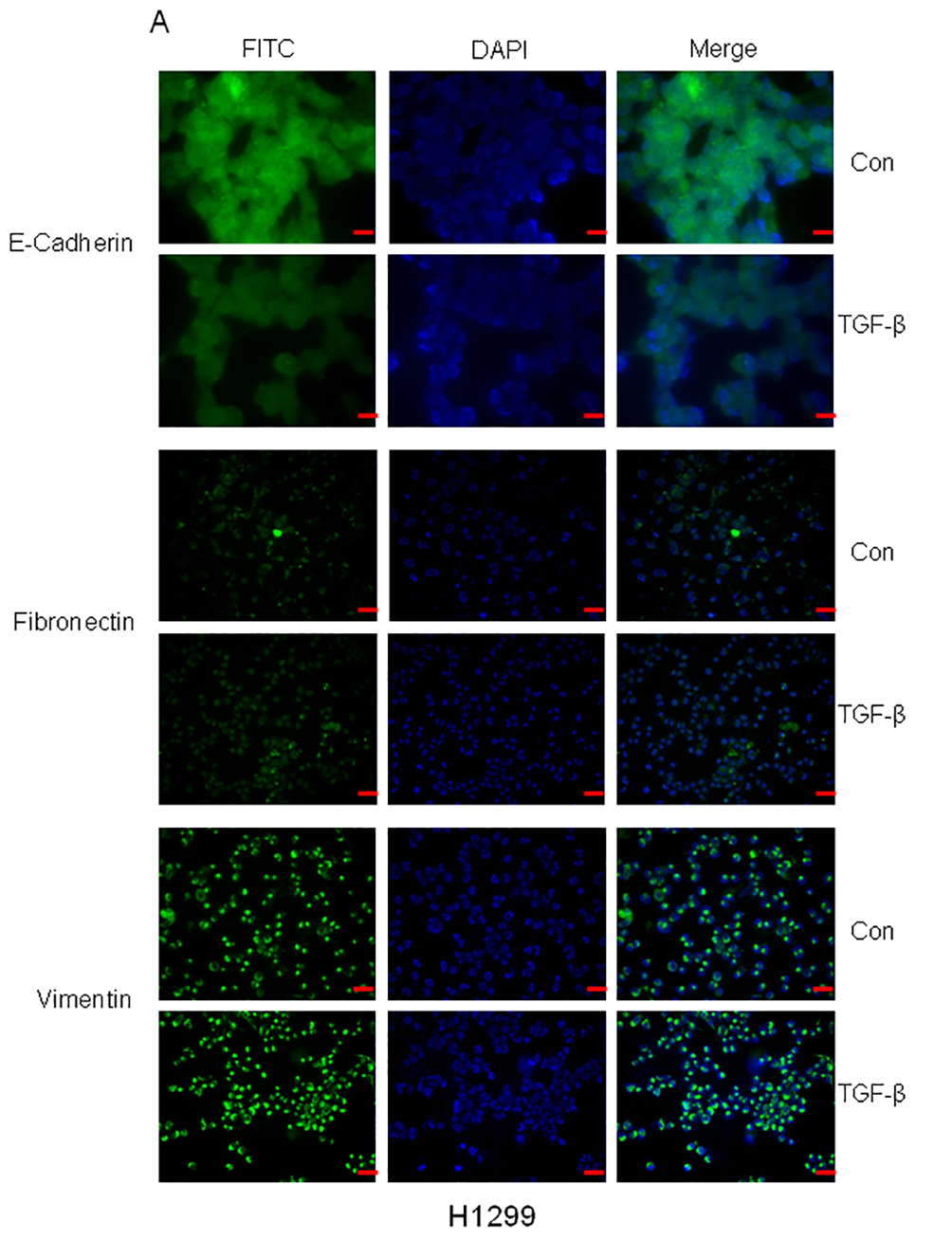

Supplement: Additional file 5: Figure S3. — (A) IF staining was performed for E-cadherin, fibronectin, and vimentin in H1299 cells treated with 10 ng/ml TGF-β1 for 3 days. (TIF 3869 kb) [file 13046_2016_450_MOESM5_ESM.tif]

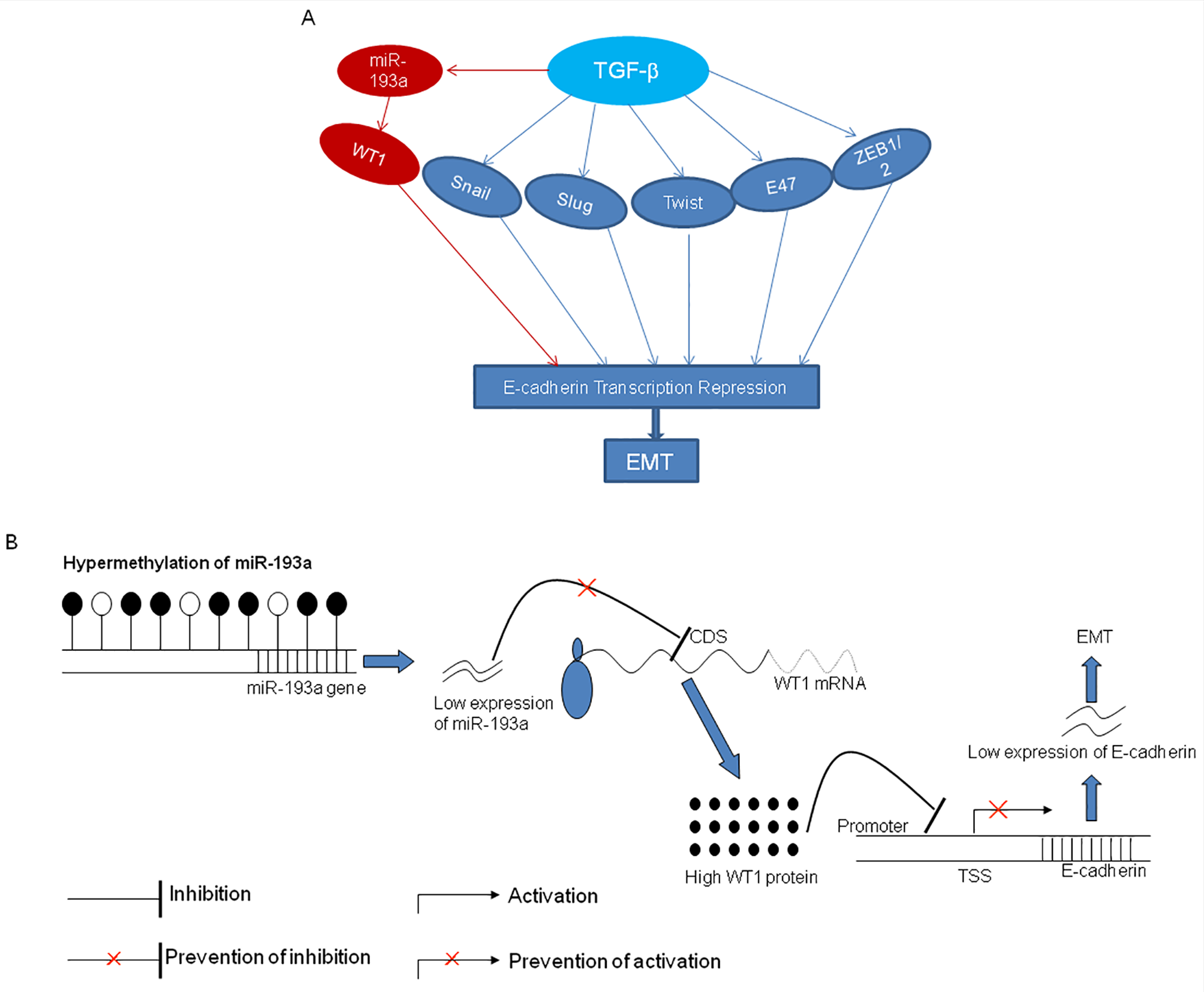

Supplement: Additional file 6: Figure S4. — (A) A schematic representation of TGF-β1-induced inhibition of E-cadherin through miR-193a-WT1 axis. (B) A schematic demonstration of DNA hypermethylation of miR-193a in lung cancer cells. Hypermethylation of miR-193a leads to the low expression of miR-193a, which loses the ability to inhibit WT1 expression. High expression of WT1 contributes to EMT through decreasing the expression of E-cadherin. (TIF 13002 kb) [file 13046_2016_450_MOESM6_ESM.tif]
